# TransEffiDet: Aircraft Detection and Classification in Aerial Images Based on EfficientDet and Transformer

**DOI:** 10.1155/2022/2262549

**Published:** 2022-04-21

**Authors:** Yanfeng Wang, Tao Wang, Xin Zhou, Weiwei Cai, Runmin Liu, Meigen Huang, Tian Jing, Mu Lin, Hua He, Weiping Wang, Yifan Zhu

**Affiliations:** ^1^College of Systems Engineering, National University of Defense Technology, Changsha 410082, China; ^2^School of Artificial Intelligence and Computer Science, Jiangnan University, Wuxi 214122, China; ^3^Graduate School, Northern Arizona University, Flagstaff, AZ 86011, USA; ^4^AiTech Artificial Intelligence Research Institute, Changsha 410000, China; ^5^College of Sports Engineering & Information Technology, Wuhan Sports University, Wuhan 430079, China

## Abstract

In recent years, analysis and optimization algorithm based on image data is a research hotspot. Aircraft detection based on aerial images can provide data support for accurately attacking military targets. Although many efforts have been devoted, it is still challenging due to the poor environment, the vastness of the sky background, and so on. This paper proposes an aircraft detection method named TransEffiDet in aerial images based on the EfficientDet method and Transformer module. We improved the EfficientDet algorithm by combining it with the Transformer which models the long-range dependency for the feature maps. Specifically, we first employ EfficientDet as the backbone network, which can efficiently fuse the different scale feature maps. Then, deformable Transformer is used to analyze the long-range correlation for global feature extraction. Furthermore, we designed a fusion module to fuse the long-range and short-range features extracted by EfficientDet and deformable Transformer, respectively. Finally, object class is produced by feeding the feature map to the class prediction net and the bounding box predictions are generated by feeding these fused features to the box prediction net. The mean Average Precision (mAP) is 86.6%, which outperforms the EfficientDet by 5.8%. The experiment shows that TransEffiDet is more robust than other methods. Additionally, we have established a public aerial dataset for aircraft detection, which will be released along with this paper.

## 1. Introduction

Analysis and optimization algorithm based on image data is a hot issue in recent years. Image processing is not only used in the civil field, but also widely used in the military field. In the military field, aerial images and remote sensing images are used to detect aircraft objects in military bases and airports in war. It is of great significance to intelligence deployment and strategic deployment. Through the acquired images, the commander can quickly and accurately understand the number of enemy aircraft on the battlefield and the take-off and landing situation. Location distribution can provide a strong information security guarantee for the follow-up operational decision and play an essential role in winning the war [1]. Therefore, aircraft detection in images is very popular in the field of military research. In addition, the classification of military and civil aircraft is also very important, which may reduce unarmed civilian casualties. These tasks must be reliable to automate site analysis, especially to export alerts corresponding to abnormal events. Therefore, how to accurately detect and classify aircraft in aerial images has high research value.

Traditional object detection requires manual feature extraction, and classifiers are designed and trained for specific detection objects. It is difficult for this kind of method to obtain robust solid features and it is very sensitive to external environmental noise, so it has significant limitations in engineering applications.

Object detection technology based on convolution neural networks is developing rapidly with the development of hardware. The two-stage method which is represented by R-CNN [2] and the single-stage method which is represented by SSD and YOLO [3] are two mainstream frameworks of object detection techniques based on convolution neural networks.

In recent years, many scholars have optimized and improved the internal backbone network, anchor design, region feature coding, and other submodules based on the two frameworks, which effectively enhance the performance of the object detection method. Some researchers have also proposed an object detection framework based on object key points [4] and have made remarkable achievements in each big data set.

However, it still faces many challenges for aircraft detection in aerial images, for example, the poor image quality due to poor environment, similar shapes of different types of aircraft, and the vastness of the sky background. The factors require more robust technologies and systems that can reliably detect and classify aircraft using characteristics. Artificial intelligence (AI) and Deep Learning (DL) can play an important role. The AI system, which is usually based on the DL, can automatically detect the aircraft, thereby enhancing military situational awareness [5].

In this paper, a robust aircraft detection method is proposed to solve the aircraft detection problems. This method is a hybrid solution based on EfficientDet and Transformer. We improved and optimized the EfficientDet algorithm by using Transformer, which models long-range dependency for feature maps, and then a feature fusion module is proposed to effectively capture both the long-range and short-range context generated by the Transformer module and efficient backbone. In addition, most aircraft detection methods are to detect targets in remote sensing images. However, it is significantly essential to detect aircraft in aerial images. Therefore, we explore aircraft detection in aerial images in this paper.

The novelty can be summarized into the following four points:

1. Create a hybrid structure by combining the power of CNNs with Transformers to extract multiscale and multidimensional feature representations. In this way, the long-range dependency of features is modeled.
2. Design a feature fusion module which can fuse features extracted from different deformable Transformer layers, so that the backbone can fuse global context information and abundant local information effectively.
3. Use the deformable attention modules to reduce the computational complexity. Therefore, our method can handle higher dimensional feature maps.
4. Obtain superior performance on the task of aircraft detection in aerial images. In addition, it is a challenging and labor-intensive task to label all the aircraft in an image. To promote the development of aircraft detection in aerial images, we will release this dataset along with this paper, which contains five types of aircraft: armed helicopters, bombers, fighter jets, early warning aircraft, and passenger aircraft, with a total of 2558 images.

## 2. Related Work

### 2.1. Traditional Machine Learning Method

Traditional machine learning methods mainly implement object detection through the following steps: constructing a training data set, region extraction, feature design and extraction, feature processing, similarity measurement selection, classifier design, training, and detection.

The main limitations of traditional machine learning methods are region extraction, feature extraction, feature processing, and classifier design.

In the search strategy of candidate region extraction, the common methods are the sliding window method, such as [6]. Feature extraction refers to feature fusion and dimension reduction. The feature extraction of the object essentially maps the high-dimensional information to low-dimensional feature space, which becomes the basis of object detection [7]. There are generally two methods for feature processing: feature fusion [8] and feature dimensionality reduction [9].

### 2.2. Artificial Neural Network

The neural networks do not need to manually design features and automatically extract features from samples through a trained network. Using only a single feature as a feature extractor is inefficient and cannot meet the needs of rapid detection in aircraft object detection tasks, and the deviation and asynchrony between different feature extractors will reduce the effectiveness of the training process [10]. Therefore, more research focuses on establishing end-to-end networks. Deep learning research has continued to develop in object detection field. These methods with powerful feature representation capabilities regards object detection as the classification problem of regions of interest using deep features, uses deep network architecture to obtain image features from input data automatically, and classifies images at the output layer. These feature maps extract rich semantic features and have strong feature representation capabilities.

According to the usage of the data set, the network structure, and different application scenarios, deep learning has derived a variety of methods, such as CNN-based image object detection network, Deep Belief Network (DBN) [11], etc.

### 2.3. Optimization of Object Detection Framework

The object detection framework generally includes backbone network, neck connection layer, anchor, region feature coding, classification and location head, loss function, and other submodules. In addition, different models have their unique submodules. The performance of the object detection method can be effectively improved using reasonable optimization.

Residual structure [12], which is used to increase network depth, can effectively improve accuracy of convolution networks, but the number of parameters increases exponentially. For this reason, Xie et al. [13] integrated the residual structure with the Inception structure [14]. The Inception structure increases the depth and width of the network by sharing the width direction parameters. This method improves the network accuracy while effectively controlling the growth of the number of parameters.

Optimization of Anchor Design: Anchors are rectangular boxes with different sizes and proportions generated in each grid on the feature graph. The single-stage detection framework generates the object boundary box directly based on the anchors, and the two-stage detection framework obtains the candidate box by fine-tuning the positive anchors.

Anchor frames with different scales are suitable for detecting different objects. For this reason, Zhu et al. [15] proposed an anchor frame design strategy based on step size reduction. The high-resolution feature image has a small receptive field and is used to detect small-scale objects. To prevent missed detection, the step size generated by the anchor frame should be reduced to increase the density of the anchor frame. Xie et al. [16] proposed a dimensionally decomposable region recommendation network. This method decomposes the anchors in the dimension, thus effectively solving the detection of proportional special objects.

To optimize the limitations of anchor-based methods in allocating positive and negative samples and dealing with multiscale problems, many scholars have proposed a target detection model without anchors.

Most of these models carry out pixel-level classification and regression on the different scale feature images to replace the anchor box. Tian et al. [17] first calculate the position of each point on the feature map that maps back to original images, then distribute the different samples, and define the center degree to reduce the fractional weight when predicting the edge position of the instance frame. As a result, the influence of the low-quality prediction box on the detection results is suppressed and the detection performance of the model is improved.

Optimization of nonmaximum suppression algorithm: In object detection, nonmaximum suppression means that, in the forward reasoning stage, the highest-confidence candidate box is selected as the final result. Besides, the surrounding candidate boxes whose intersection and union ratio are greater than the threshold are eliminated. For the same detected object, this method can eliminate other nonoptimal candidate results and avoid relocation.

To solve the issues, Bodla et al. [18] proposed an algorithm for soft suppression of SoftNMS. It reduces the confidence of the first *n* nonoptimal candidate boxes instead of eliminating them directly. The confidence of the candidate box is not strongly related to the intersection and union ratio, only considering that the classification confidence is one-sided. He et al. [19] improved the soft suppression algorithm and incorporated the location confidence into it to indicate the credibility of the coincidence of the current candidate box and the instance box. It models the candidate box and the instance box, respectively, and uses KL divergence to measure the distance. In addition, Liu et al. [20] designed a subnetwork containing only the fully connected and convolution layer, which is used to determine whether the nonoptimal candidate box whose intersection and merge ratio is greater than the threshold predicts the same target as the optimal candidate box and retains the nonoptimal candidate box with different detection targets, so as to effectively avoid the disadvantages of the traditional methods.

Optimization of positive and negative sample sampling algorithm: There are a large number of anchor frames on large-scale feature maps, and most of the negative sample anchor boxes provide similar gradient information, so all of them are used in classification and regression training to waste computational resources. Therefore, sampling all anchor boxes is required. Only some of them are selected to participate in the training.

Since the number of positive anchor boxes is much smaller than that of negative anchor boxes, it is easy to cause the imbalance between positive and negative training samples by sampling randomly in the whole world. The SSD model sorts the negative samples according to the confidence error and updates the model for the difficult negative samples with low confidence.

Different from the SSD model, Shrivastava et al. [21] select complex negative samples online according to the loss of input samples. They extended the two-stage detection framework, designed another RoI network to calculate the loss of input samples, and reduced the order of input loss, and selected the first *n* negative samples with the largest loss for model training. The advantage of using input loss as a standard to measure the difficulty of sample learning is that it can consider both the difficulty of classification and regression. Inspired by the above research, Yu et al. [22] adopted a similar method to optimize the positive and negative sample sampling of the single-stage detection framework. It directly filters simple samples and only backpropagates the *k* samples with the largest loss to update the network parameters. The difficult negative sample can also be represented by the intersection and union ratio with the instance box.

## 3. Material and Method

This paper proposes an improved object detection and classification architecture named TransEffiDet, which is based on EfficientDet [23] and Transformer [24]. The deep neural network is helpful to extract high-level information. However, gradient vanishing is prone to occur simply by deepening the number of layers in the network. In other words, the EfficientDet cannot model the long-range dependence, because of the network architecture. However, the proposed TransEffiDet has the improved Deformable Transformer module. With the proposed feature fusion strategy, the Transformer can better model the long-term dependence for the generated features, and the performance of TransEffiDet is improved.

### 3.1. Experimental DATA

**Figure 1:**
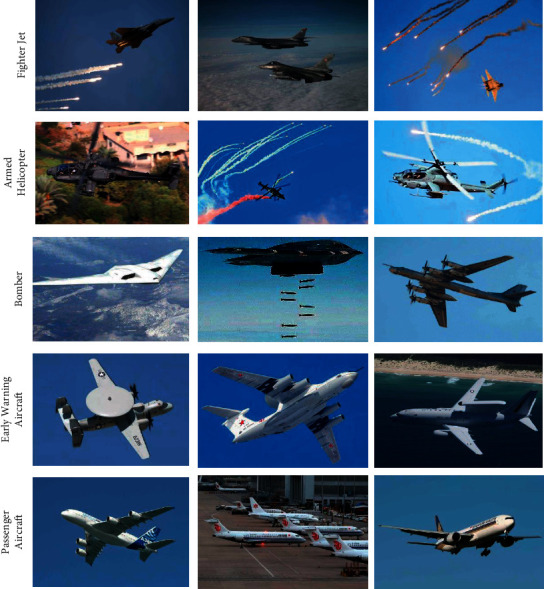
The MADAI dataset we released. MADAI contains four types of military aircraft (fighter jets, armed helicopters, bombers, and early warning aircraft) and one type of civil aircraft (passenger aircraft).

To promote the development of aircraft detection in aerial images, we have established a data set, named Military Aircraft Detection in Aerial Images (MADAI), containing four types of military aircraft: fighter jets, armed helicopters, bombers, and early warning aircraft, as shown in Figure 1. In addition to military aircraft, we have added passenger aircraft to the dataset, which helps to distinguish military and civilian aircraft, thereby reducing civilian casualties. Therefore, there are five types of aircraft in the MADAI dataset: fighter jets, armed helicopters, bombers, early warning aircraft, and passenger aircraft. There are a total of 2558 images in this dataset. The typical resolutions are 1600 × 1024, 3500 × 2280 pixels.  Table 1 shows the number of training and testing images. The MADAI contains the images and the ground truth. The ground truth, annotated by three experienced experts, indicates the type and location of the aircraft. The MADAI dataset can not only be utilized to measure the accuracy of the aircraft detection algorithm, but also provide ideas for subsequent object strikes in the military field. This dataset and annotations can be obtained from https://github.com/wangyanfeng231/TransEffiDet.

**Figure 2:**
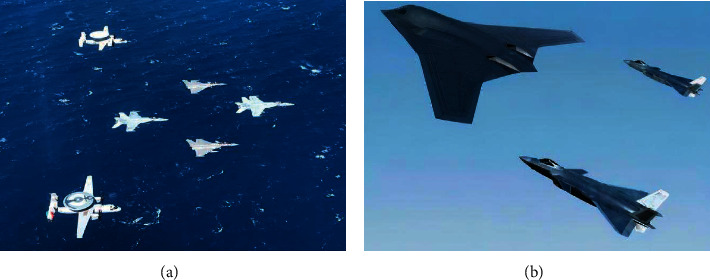
Joint operations of different types of military aircraft. (a) Joint military operations of early warning aircraft and fighter jets. (b) Joint military operations of bombers and fighter jets.

Moreover, each image in our dataset may contain multiple types of aircraft rather than just one type, and each image contains a different number of aircraft objects. This is consistent with the actual military use because military aircraft usually operates jointly, such as early warning aircraft and fighters, bombers and fighters, as shown in Figure 2.

### 3.2. TransEffiDet Architecture

**Figure 3:**
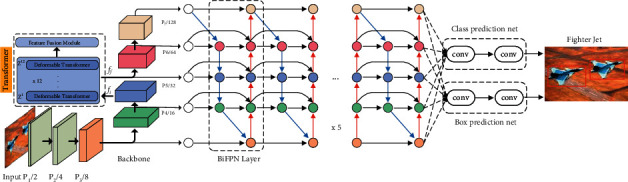
Diagram of TransEffiDet. EfficientDet [23] is backbone; BiFPN is feature extraction network. The Transformer modules are added between the P5 and P6 layers.

The architecture of TransEffiDet is shown in Figure 3. This architecture follows the paradigm of single-stage objection detection methods.

**Figure 4:**
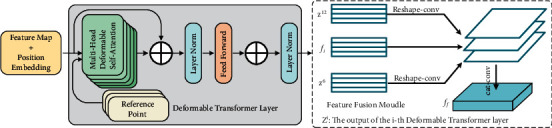
Diagram of Transformer and feature fusion module.

We employ EfficientDet as the backbone network, which can efficiently fuse the different layer multiscale feature maps. BiFPN, which is detailed in Section 3.3, is unitized as the feature network. We take the 3–7 level features of the network and repeatedly apply feature fusion model BiFPN to extract the context features. Moreover, to model the long-term dependence of different layer features, we add a 12x deformable Transformer between the P5 and P6. The diagram of Transformer and feature fusion module are shown in Figure 4. Finally, object class is produced via a class prediction net, and bounding box predictions are generated by feeding these fused features to box prediction net. Similar to [25], in all levels of features, the class prediction net and the box prediction net shared the same weights.

### 3.3. BiFPN

**Figure 5:**
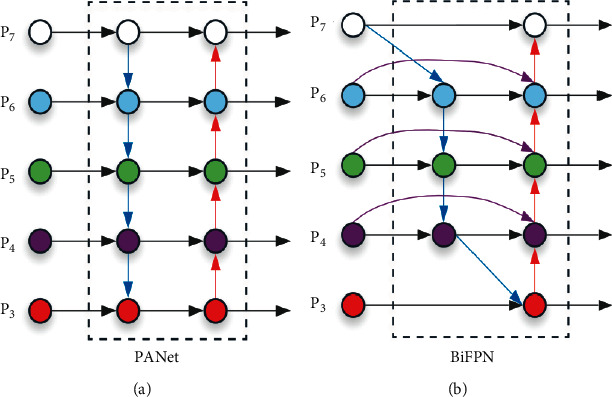
PANet and BiFPN architectures. (a) The PANet. (b) The BiFPN used in TransEffiDet.

BiFPN has made a number of optimizations for multiscale connections to improve the efficiency. First, compared with the PANet [26] (Figure 5(a)), the node with only one input is deleted, because these nodes have little benefit to the fusion of different level features. Second, in each layer, an additional edge is added liked ResNet, which connects the input and output. This setting can integrate more features. Third, the BiFPN is repeated to produce more high-level features. See reference [23] for details.

We used the fast normalized fusion. Since the network does not have the Softmax in fast normalized fusion, it is more efficient.

### 3.4. Transformer Module

Since the intrinsic locality of convolutional networks, convolutional neural networks cannot effectively model the long-range dependency between pixels. Therefore, deformable Transformer encoder (DeTrans-encoder) layer is constructed to map long-term dependency between pixels to effectively extract local as well as global semantic information. The deformable Transformer introduces a deformable self-attention mechanism (DMSA) which can reduce the hardware requirement. DeTrans-encoder layer consists of an Input-to-Sequence Transformation (IST) layer and a deformable Transformer layer.

IST layer is necessary to first convert the feature map generated by the CNN-encoder {P5} into a one-dimensional sequence, because the Transformer only processes the input by a sequence form. However, directly converting a 3D feature map into a 1D sequence will result in the loss of spatial features. Hence, the sine and cosine functions are used to obtain a 3D position-encoded sequence, shown as follows:

(1) $$\begin{array}{c} {\text{PE}}_{\left(\text{local},2i\right)}=\sin\left(\frac{\text{local}}{10000^{2i/d_{\text{model}}}}\right),\\ {\text{PE}}_{\left(\text{local},2i+1\right)}=\text{con}\left(\frac{\text{local}}{10000^{2i/d_{\text{model}}}}\right), \end{array}$$

where *local* is the location, and *d*_model_ is the dimension of the CNN feature map. We obtain the input sequence of DeTrans-encoder by summing the extracted features with the position-encoded sequence.

#### 3.4.1. DMSA Layer

The self-attention mechanism of the Vanilla Transformer focuses on all possible positions of the feature map, which causes the network to converge more difficult. In addition, the computational and spatial complexities of the self-attentive mechanism grow squarely with the image size, making it difficult to handle high-resolution features. To address these issues, we use a deformable self-attentive mechanism that can significantly reduce the complexity by focusing on only a small number of sampled locations instead of all locations of the feature map based on automatically determined reference points [27].

We have an input feature map *f*_*i*_ ∈ *R*^*C*×*H*×*W*^, let *q* be a query element with a feature value *z*_*q*_ ∈ *R*^*C*^, and *p*_*q*_ is a 2-d reference coordinate point. The deformable attention feature can be calculated as

(2) $$\begin{array}{c} \text{DeformAttn}\left(z_{p},p_{q},x\right)=\sum_{m=1}^{M}W_{m}\left[\sum_{k=1}^{K}A_{mqk}\cdot W_{m}^{\prime }x\left(p_{q}+\Delta p_{mqk}\right)\right], \end{array}$$

where *m* is the attention heads, *k* is the sampled keys, and *K* represents the total sampled key number, where *K* ≪ *D* × *H* × *W* thereby not only speeding up the convergence but also significantly reducing the computational and spatial complexities. The Δ*p*_*mqk*_ ∈ [0,1] and *A*_*mqk*_ (∑_*k*=1_^*K*^*A*_*mqk*_=1) are the sampling offset and attention weight of the k-th sampling point in the m-th attention head, respectively. For a point *p*_*q*_+Δ*p*_*mqk*_ that does not fall in integer coordinate positions, we use bilinear interpolation to obtain the final result *x*(*p*_*q*_+Δ*p*_*mqk*_). Δ*p*_*mqk*_ and *A*_*mqk*_ are obtained by performing a linear transformation of *z*_*q*_.

#### 3.4.2. Deformable Transformer Layer

Based on the above image-sequence transformation and the deformable attention mechanism, the attentional feature map can be obtained, and the deformable Transformer layer can be obtained by a feedforward network. Layer normalization operations are performed after both the attention layer and the feedforward layer, and skip connections are used in each layer to prevent the gradient from vanishing. The deformable Transformer layer is obtained by cascading multiple deformable Transformers.

#### 3.4.3. Feature Fusion Module

**Figure 6:**
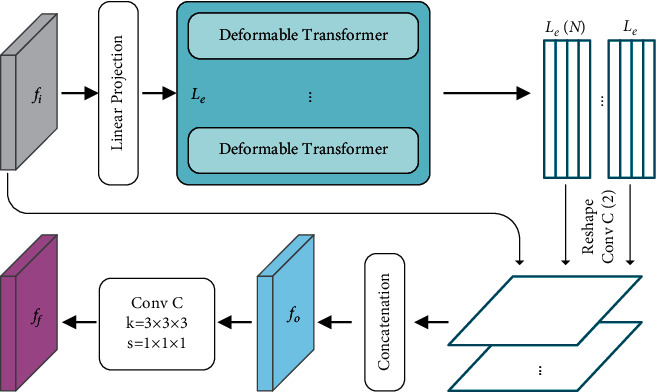
The detailed dimension changes for Transformer.

Figure 6 shows the detailed dimension changes for the Transformer, which is designed to solve the dimensional inconsistency between feature maps of the Transformer and CNN backbone. Firstly, the feature map *f*_*i*_ of level 5 (P5) of CNN-encoder is flatten into a 1D sequence. Then the flattened sequence is sent to the Deformable Transformer introduced above. The output of the Transformer is also the 1D sequence, whereas the CNN backbone receives a 2D feature map as the input. Therefore, we reshape the 1D sequence to the original dimension of P5. To effectively fuse the global context generated by different Transformer layers and local information extracted by EfficientDet backbone, we designed a feature fusion module to capture both the global and local context. Specifically, assuming that the Transformer module is composed of *L*_*e*_(*L*_*e*_=12) layers, we take out N features uniformly {*Z*^*n*^}(*n* ∈ {*L*_*e*_/*N*, 2*L*_*e*_/*N*,…, *NL*_*e*_/*N*}) with a step length *L*_*e*_/*N* as the input of the feature fusion module, and N sets to 2 in this paper. For each feature sequence outputted by deformable Transformer layer, we first reshape it into a 2-dimensional feature map with the same size of P5. And then, the convolutional operation is employed to each 2-dimensional feature map, and the output channel of this convolution is halved. In order to combine the global context information modeled by Transformer and the rich semantic information extracted by EfficientDet backbone, we concatenate all the feature maps with halved channels and the input feature map P5 to obtain a feature map *f*_*o*_. Finally, the feature map *f*_*o*_ is fed to a convolutional layer and the same channel to perform adaptive feature calibration to obtain the final fusion feature *f*_*f*_.

In this paper, the output of the sixth and twelfth Transformer layers and *f*_*i*_ are used to produce the input of the layer {P6}, which can better obtain the characteristics of different layers and keep the balance between the calculation and the efficiency. Specifically, these two resized feature maps are concatenated to produce *f*_*o*_. Finally, the feature *f*_*f*_ is obtained by a convolution function.

### 3.5. Implementation Details

#### 3.5.1. Data Augmentation

We used data augmentation method to realize different feature learning by adding different feature variables to the image. To expand the training set while preserving the basic features, data augmentation is carefully applied to get some new images. Various random changes were included, including movement, rotation, zooming, and horizontal/vertical flip.

#### 3.5.2. Pretrained Weights

In EfficientDet, the networks are pretrained using the ImageNet. Following this tradition, all models of TransEffiDet are pretrained on ImageNet. Then it is fine-tuned on our datasets. Since the high-order features are learned on the ImageNet, which is different from the MADAI dataset, we retrained some convolution blocks to fine-tune the weight of the classification task.

#### 3.5.3. Other Details

The images are resized to 768 × 768 pixels to reduce memory requirements during training. The TransEffiDet architecture is implemented using PyTorch. We used SGD optimizer with 0.9 momentum. The validation set is 25% of the training data. To facilitate a fair comparison, the metrics provided in this paper correspond to the best performance in the validation and training dataset, so the performance of the proposed method is not, in any way, optimized for the test datasets. The source code is publicly available at https://github.com/wangyanfeng231/TransEffiDet.

## 4. Results and Discussion

### 4.1. Experimental Results

In this section, the performance of TransEffiDet is evaluated on our dataset. Both EfficientDet and the proposed TransEffiDet have been retrained using the MADAI dataset. Detection performance measures are shown in Table 2. Compared with EfficientDet, the Average Precisions (AP) of Bomber, Fighter, and Armed Helicopter are improved by 17.7%, 8.4%, and 2.5%, respectively. In total, the mean Average Precision (mAP) is improved by about 5.8%. From the quantitative results, we can see that the performance of TransEffiDet is better than EfficientDet.

**Figure 7:**
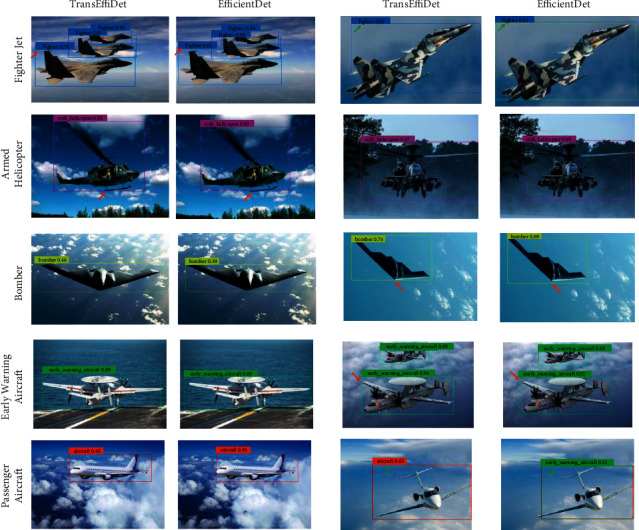
Examples of aircraft detection results in the MADAI dataset.

The aircraft detection results of TransEffiDet and EfficientDet are illustrated, as shown in Figure 7. Each type of aircraft is marked with different color boxes, and the numbers next to the boxes represent confidence. We can see that most of the aircraft are accurately detected, especially the early warning aircraft. This shows the advantage of this method; that is, it can detect aircraft accurately. Compared with EfficientDet, TransEffiDet can achieve more accurate detection and the detected box can achieve better precision (red arrows in Figure 7). The detection box produced by EfficientDet is larger or smaller than the real object, which leads to lower accuracy. Moreover, the method EfficientDet will produce some false positives of aircraft. This is mainly because some types of aircraft are similar such as bomber and fighter, early warning aircraft, and passenger aircraft, and so on. Therefore, it is hard for the network to detect these similar objects. However, the proposed TransEffiDet can handle this problem well, because the Transformer can provide the long-term relationship and further make the network focus on the aircraft's features.

### 4.2. Ablation Study

The ablation study is conducted on all test datasets to illustrate the effectiveness of the feature fusion module, as shown in Table 3. We explore the fusion i.e., concatenation (Cat), add (Add), of the input and Transformer outputs of different layers (*Z*^4^, *Z*^6^, *Z*^8^, *Z*^12^) to obtain the optimal fusion feature representation. All outputs *Z*^*l*^ of the Transformer are fed into a convolution layer (kernel size 3 × 3) with (*∗*/half) and without the feature channels halved operation. *∗* represents the input and the outputs of different Transformer layers.

We investigated the contribution of the outputs of different Transformer layers and the input. From the results of Models 1, 2, and 8 in Table 3, we can see a clear trend that the more the feature maps are added, the better the performance is. Compared with Model 1, Model 2 significantly improves the performance by adding a feature map of the Transformer middle layer. The further improved performance was obtained by Model 4 by introducing the input into the final fusion feature.

Furthermore, to explore the influence of the feature fusion way, Models 3, 4, and 5 were built. We can see that the concatenation fusion way can obtain relatively good detection results. This is because simple addition cannot integrate different feature maps well. Finally, we built Models 6 and 7 to further demonstrate the effectiveness of the proposed feature fusion module. Combining the long-term modeling capabilities of the Transformer and the abundant local information of the feature maps results in the good performance of Model 8.

## 5. Conclusion

This paper proposes an aircraft object detection method named TransEffiDet in aerial images based on EfficientDet and Transformer methods. We improved the EfficientDet object detection algorithm by combining it with the Transformer which models the long-term correlation of the features. The mAP of the proposed TransEffiDet in aerial images can reach 86.6%, which outperforms the EfficientDet by 5.8%. The experimental results show that TransEffiDet has good robustness and is more suitable for aircraft detection and classification tasks in military field than the compared methods. Additionally, we have established a public aerial dataset for aircraft detection and classification, which will be released along with this paper. In the future, we will explore the application of this method to target detection in military field, which may require faster detection speed.

In this study, our proposed method is employed to detect aircrafts, but so far it is not easy for this method to accurately detect fighter jets, bombers, and passenger aircraft. A possible explanation is that the shape features of these aircrafts are not very obvious, so the feature extraction network cannot effectively extract these features for classification. In our future work, we try to use swim-transformer to extract rich global and local features to improve detection accuracy.

## Figures and Tables

**Table 1:** The detailed numbers of each type of aircraft.

	Bomber	Passenger aircraft	Early warning aircraft	Fighter	Armed helicopter	Total
Training	348	378	318	481	494	2019
Testing	100	97	97	135	110	539
Total	448	475	415	616	604	2558

**Table 2:** The performance measures for the EfficientDet and TransEffiDet.

	AP					mAP
	Bomber	Early warning aircraft	Fighter	Armed helicopter	Passenger aircraft	
TransEffiDet	**55.4**	**98.9**	**84.9**	**98.2**	**95.3**	**86.6**
EfficientDet	37.7	98.8	76.5	95.7	95.2	80.8

**Table 3:** The ablation study of the proposed method on all test datasets.

Models	Ablation type										mAp (%)
	Input	Input/half	*Z* ^6^	*Z* ^6^/half	*Z* ^12^	*Z* ^12^/half	*Z* ^4^, *Z*^8^, *Z*^12^/half	Cat	Add	Cat/Add	
Model 1					**√**						76.46
Model 2				**√**		**√**		**√**			81.03
Model 3	**√**		**√**		**√**				**√**		82.67
Model 4	**√**		**√**		**√**			**√**			82.86
Model 5	**√**			**√**		**√**				**√**	82.84
Model 6		**√**		**√**		**√**		**√**			81.67
Model 7	**√**						**√**	**√**			85.06
Model 8 (proposed)	**√**			**√**		**√**		**√**			**86.55**

## Data Availability

Data are available at https://pan.baidu.com/s/11UORs4eaKKPZNscIrtbISg; Extraction code: data.
